# Synthesis, Characterization and Biological Activities of Biopolymeric Schiff Bases Prepared with Chitosan and Salicylaldehydes and Their Pd(II) and Pt(II) Complexes

**DOI:** 10.3390/molecules22111987

**Published:** 2017-11-16

**Authors:** Hellen Franciane Gonçalves Barbosa, Maha Attjioui, Ana Paula Garcia Ferreira, Edward Ralph Dockal, Nour Eddine El Gueddari, Bruno M. Moerschbacher, Éder Tadeu Gomes Cavalheiro

**Affiliations:** 1 Instituto de Química de São Carlos, Universidade de São Paulo, Av. Trabalhador São Carlense, 400, São Carlos 13566-590, SP, Brazil; hellenbarbosa@usp.br (H.F.G.B.); anagarcia@iqsc.usp.br (A.P.G.F.); 2 Institute of Plant Biology and Biotechnology (IBBP), Westfälische Wilhelms-Universität Münster, Schlossplatz 8, 48143 Münster, Germany; m_attj01@uni-muenster.de (M.A.); guedari@uni-muenster.de (N.E.E.G.); 3 Departmento de Química, Universidade Federal de São Carlos, Via Washington Luis, Km 235, São Carlos 13560-900, SP, Brazil; erdockal@gmail.com

**Keywords:** chitosan, Schiff bases, complexes, antimicrobial, antitumor

## Abstract

In an attempt to enhance chitosan biological activities, biopolymeric Schiff bases of chitosan and different salicylaldehydes and their palladium(II) and platinum(II) complexes were synthesized and tested. The chemical structures of these derivatives were characterized using ^1^H-NMR, FTIR spectroscopy and XPRD. Thermal analysis was done through TGA/DTG-DTA. Electronic absorption spectra and surface morphologies were analyzed by SEM-EDAX. Chitosan and its derivatives were evaluated for their in vitro antimicrobial activity against two common bacterial and fungal plant pathogens *Pseudomonas syringae* pv. *tomato* and *Fusarium graminearum*, respectively, and for their antitumor activity against a human breast cancer cell line (MCF-7). It was found that, compared to the nonmodified chitosan, chitosan modified with Schiff bases and their complexes was highly toxic against the MCF-7 cell line and had antibacterial effects against *P. syringea*. However, the modified chitosan derivatives had less pronounced antifungal effects against *F. graminearum* compared to the nonmodified chitosan, suggesting different modes of action.

## 1. Introduction

Chitosan is a linear aminopolysaccharide consisting of 2-deoxy-d-glucosamine and 2-deoxy-*N*-acetyl-d-glucosamine units linked by glycosidic β-(1→4) bonds. This biopolymer is an abundant cationic polysaccharide obtained by the deacetylation of chitin, a polysaccharide widely distributed in nature [1]. Chitosan presents important physiological properties such as biocompatibility, biodegradability, and nonallergenic qualities as well as interesting biological activities such as antifungal, antibacterial, antiviral and antitumor activities [2,3,4,5]. These properties make chitosan very attractive for a variety of applications in the pharmaceutical, medical, agricultural, food and cosmetic industries and as a treatment for waste water [6,7,8].

Recently, the attention of researchers has been drawn toward the chemical modification of chitosan in order to enhance its biological activities [9]. Common chemical reactions are acylation, carboxymethylation, cyanoethylation, phosphorylation and Schiff base condensation [9,10,11,12,13,14,15]. For example, chitosan can be chemically modified by condensation of the deacetylated fractions of the polymer with aldehydes in a homogeneous phase; this condensation is carried out between primary amines and aldehyde groups to form corresponding Schiff bases [16,17].

A very important class of Schiff bases is made up of compounds derived from aromatic aldehydes, as these compounds efficiently complex with many metal ions that can be used as heterogeneous catalysts [18,19,20]. Additionally, the Schiff base’s azomethine linkage (–C=N) is an essential structural requirement for biological activities, including antibacterial, antifungal and antitumor activities [9,12,13,14]. Moreover, desired properties can be adapted by choosing appropriate amines or carboxylic substituents in the amine group [14,18,21], especially when a functional group like –OH or –SH is present close to the azomethine group [10,16,20]. In addition, it has been shown that a large number of Schiff base complexes such as platinum and palladium possess antibacterial, antiviral, antimicrobial, anti-inflammatory and anticancer activities [22,23].

**Scheme 1:**
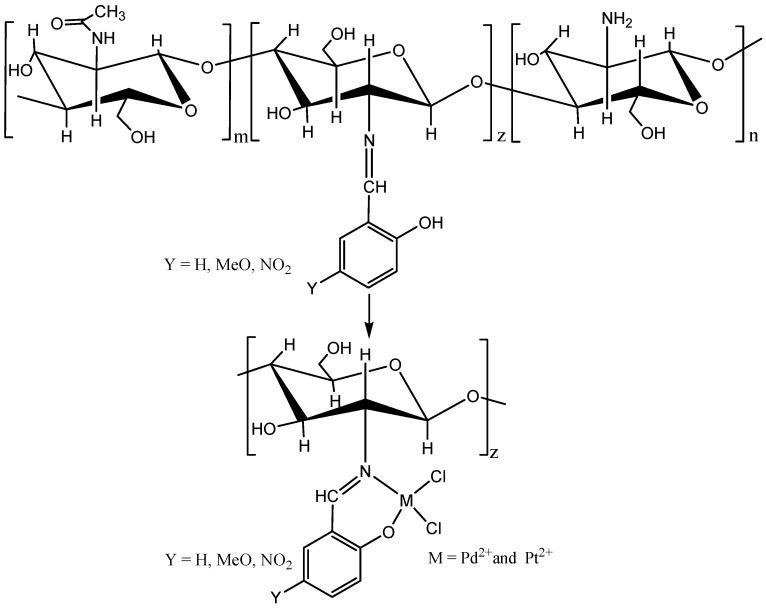
Structural representation of the ligands *ortho-hydroxyaryl* Schiff base of chitosan, palladium and platinum metal complexes.

Therefore, to potentially improve the bioactivity of chitosan, in the present study, chitosan was reacted with salicylaldehyde (2-hydroxybenzaldehydes) and 5-methoxy-salicylaldehyde and 5-nitro-salicylaldehyde derivatives to form biopolymeric Schiff bases; these were then used to synthesize their respective palladium(II) and platinum(II) complexes (Scheme 1).

The compounds were characterized by ^1^H-NMR, SEC-MALS-RI, FTIR, TGA/DTG-DTA, SEM/EDAX, XPRD and electronic spectral techniques. These chitosan derivatives were then tested for their biological activities including antimicrobial and antitumor activities. To our knowledge, this study represents the first report on the ortho-hydroxyaryl Schiff base of chitosan, its palladium(II) and platinum(II) complexes and their potential biological activities.

## 2. Results and Discussion

### 2.1. ^1^H-NMR Spectroscopy

**Figure 1:**
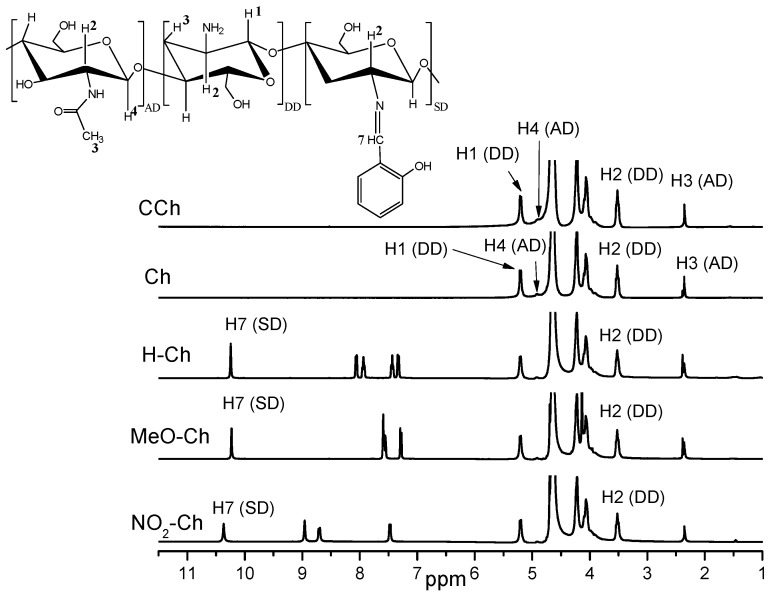
^1^H-NMR spectra of CCh chitosan, Ch chitosan and biopolymeric Schiff bases H-Ch, MeO-Ch and NO_2_-Ch. Temperature 70 °C, solvent HCl/D_2_O (1%).

The degree of deacetylation $\left(\bar{\mathrm{DD}}\right)$ of commercial chitosan (CCh) and purified chitosan (Ch) were determined from ^1^H-NMR spectra (Figure 1) as 90.1% and 90.4%, respectively. These values were determined from the ratio of areas of the hydrogen bonded to carbon 2 in the pyranose ring related to signals at 3.5 ppm (H2) and the signal in the region of 2.3 ppm (H3) attributed to the acetamido methyl hydrogens [24]. The degree of substitution $\left(\bar{\mathrm{DS}}\right)$ for biopolymeric Schiff bases formed with salicylaldehyde and 5-methoxy and 5-nitro-salicylaldehyde derivatives were calculated from the ratio between the integrated resonances of the hydrogen at carbon 7 (H7) in the imine groups at 10.3 ppm and the hydrogen at carbon 2 in the glucopyranoside ring in the region of 2.4 ppm (H2). The $\left(\bar{\mathrm{DS}}\right)$ for salicylaldehyde chitosan (H–Ch), 5-methoxy-salicylaldehyde chitosan (MeO–Ch) and 5-nitro-salicylaldehyde chitosan (NO_2_–Ch) were 51.2%, 54.3% and 48.0%, respectively. The hydrogen assignments are shown in Figure 1.

The average molecular weight ($\bar{\mathrm{Mw}}$) of CCh and Ch were measured by size exclusion chromatography coupled on line with a refractive index detector and multi-angle laser light scattering (SEC-MALS-RI). The values of $\left(\bar{\mathrm{DD}}\right)$, $\left(\bar{\mathrm{DS}}\right)$, polydispersities and ($\bar{\mathrm{Mw}}$) for CCh, Ch and the Schiff bases are shown in Table 1.

The values of mean molar mass per residue ($\bar{\mathrm{MM}}$) were determined using Equation (1). The theoretical values of molar mass were calculated using degree of acetylation, deacetylation and substitution determined by ^1^H-NMR and the values of molar mass of acetylated, deacetylated and substituted monomers.

(1) $$\bar{\mathrm{MM}}=\frac{\left({\mathrm{MM}}_{\mathrm{AM}}\times \bar{\mathrm{DA}}\right)+\left({\mathrm{MM}}_{\mathrm{DM}}\times \bar{\mathrm{DD}}\right)+\left({\mathrm{MM}}_{\mathrm{SM}}\times \bar{\mathrm{DS}}\right)}{100}$$

where *MM_AM_* is molar mass of the acetylated monomer, *MM_DM_* is molar mass of the deacetylated monomer and *MM_SM_* is molar mass of the substituted monomer. $\left(\bar{\mathrm{DA}}\right)$ is the mean degree of acetylation, $\left(\bar{\mathrm{DD}}\right)$ the degree of deacetylation and $\left(\bar{\mathrm{DS}}\right)$ the degree of substitution.

### 2.2. Infrared Spectroscopy

FTIR spectra of Ch polymer showed an N–H stretching band at 1594 cm^−1^, C=O stretching band at 1652 cm^−1^, CH_3_ symmetrical angular deformation at 1380 cm^−1^, C–N amino group axial deformation at 1424 cm^−1^, C–N amide group axial deformation at 1324 cm^−1^ and characteristic polysaccharide bands at 1155 cm^−1^ for C–O stretching from β-(1→4) glycosidic bonds [25]. FTIR spectra for biopolymeric Schiff bases H–Ch, MeO–Ch and NO_2_–Ch showed strong bands at 1631, 1638 and 1640 cm^−1^, respectively, due to stretching vibrations of C=N, characteristic of imines, which are not observed for chitosan. These bands were in agreement with the results observed by Majerz et al. (2000) and also with the theoretical study by Pajak et al. (2007) [26,27]. Characteristic bands for axial deformation of the aromatic ring C=C appear from 1500 to 1660 cm^−1^. From 800 to 675 cm^−1^, bands related to angular deformation of aromatic ring C–H was observed. In spectra of substituted chitosans, N–H stretching was superimposed onto the C=O stretching bands.

The FTIR spectrum of Schiff bases containing the nitro group NO_2_ featured strong bands at 1547 and 1341 cm^−1^ due to symmetric axial deformation. In general, compounds that have nitro groups absorb strongly at 1530–1500 cm^−1^ and weakly at 1370–1330 cm^−1^. Aromatic nitro compounds exhibit absorption in the region of 760–705 cm^−1^. In the MeO–Ch spectrum, corresponding to chitosan substituted with a methoxy group, there was a stretching band at 2833 cm^−1^ superimposed with CH_2_ and CH_3_ methoxy and aromatic groups [28]. (Figure S1 available in supplementary material). FTIR spectra of Pd(II) and Pt(II) complexes revealed that the complexation of metals in Schiff bases promoted a small displacement and the formation of new bands. The complexes’ spectra exhibited bands of low intensity, suggesting metal bonds with the oxygen (M–O). The major bands of weak intensity related to metal bonds with nitrogen (M–N), and they appeared in the region of 350 and 300 cm^−1^ and around 250 cm^−1^ [29,30]. (Figure S2 available in supplementary material).

### 2.3. Thermal Analysis

**Figure 2:**
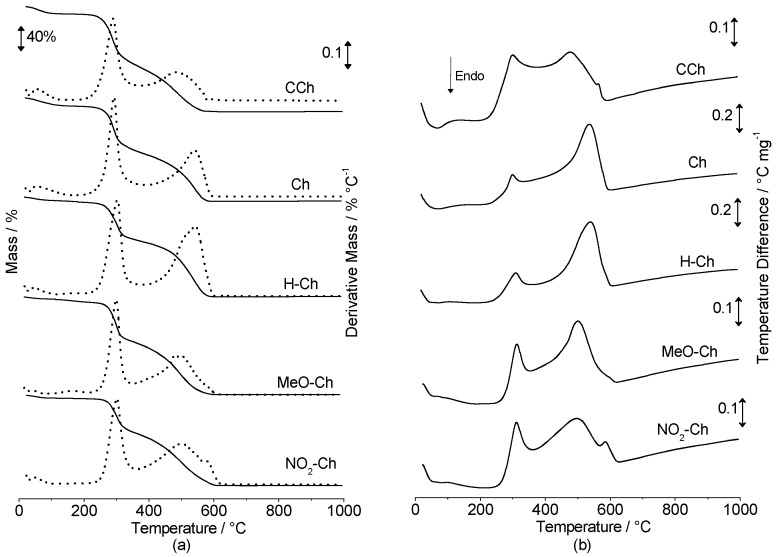
TGA/DTG (**a**) and DTA (**b**) curves of CCh, Ch, H–Ch, MeO–Ch and NO_2_–Ch, under air atmosphere, heating rate of 10 °C min^−1^, air flow rate 100 mL min^−1^, sample mass 6 mg ± 0.1, α-alumina sample holder.

TGA/DTG-DTA curves were obtained under an air atmosphere from room temperature to 1000 °C, with a heating rate of 10 °C min^−1^ for commercial and purified chitosan and also for substituted salicylaldehyde and derivatives (Figure 2). Under a dry air atmosphere, polymers have three mass loss steps. Initially, the polymers undergo a dehydration process, followed by decomposition that occurs in two stages and no residue is left after the decomposition. Table 2 summarizes the mass losses, percentage of residue and temperature range observed at each stage of the TGA curves for the samples of chitosan and its bases. These two stages of decomposition (mass loss) are related to the decomposition of organic matter for CCh and Ch. The percentage of mass loss was lower for H–Ch, MeO–Ch and NO_2_–Ch, when compared to CCh and Ch, suggesting some substitution. DTA curves exhibited three events for all compounds, the first endothermic event related to dehydration, and the other two exothermic events related to decomposition, in agreement with TGA. According to the ratios between mass losses in the first and second steps, after the initial water loss, the ratio for chitosans was close to 1.0, indicating that the mass losses in the first and second step were almost the same. For Schiff bases, there was a decrease in the value of the ratio; furthermore, the increase in mass loss during the second decomposition step suggests that modification in the substituted fraction of biopolymers takes place at higher temperatures (values are shown in Table 2). The order of thermal stability for chitosans and Schiff bases after the dehydration was Ch > CCh > NO_2_-Ch > H–Ch > MeO-Ch, revealing that chitosans have higher thermal stability then substituted chitosans.

**Figure 3:**
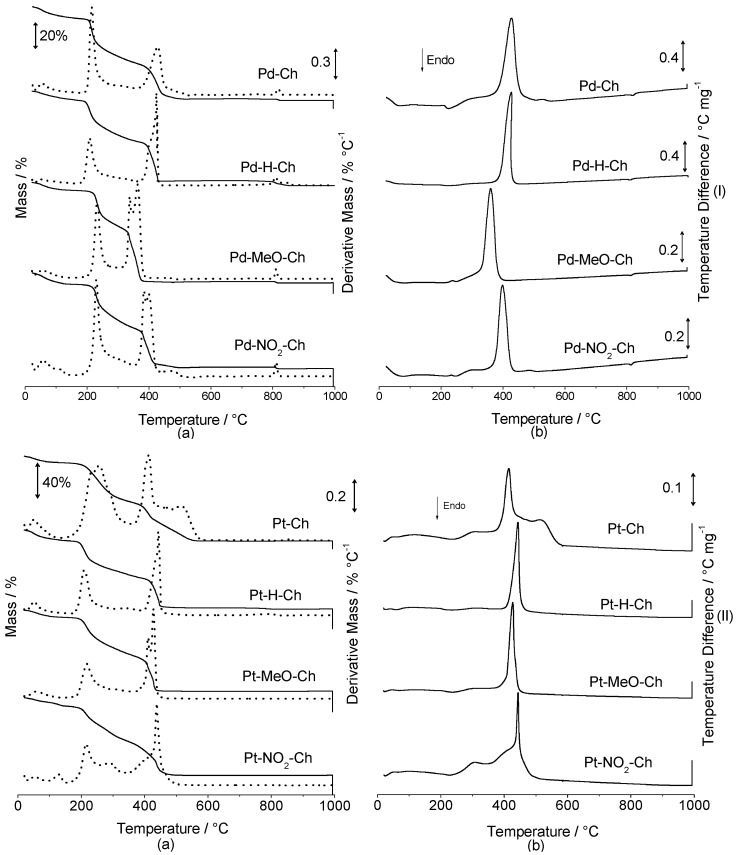
TGA/DTG (**a**) and DTA (**b**) curves of palladium complexes (**I**) Pd–Ch, Pd–H–Ch, Pd–MeO–Ch, Pd–NO_2_–Ch and platinum complex (**II**) Pt–Ch, Pt–H–Ch, Pt–MeO–Ch, Pt–NO_2_–Ch, both under an air atmosphere, heating rate of 10 °C min^−1^, air flow rate 100 mL min^−1^, sample mass 6 mg ± 0.1, α-alumina sample holder.

Palladium(II) and platinum(II) complexes as well as Schiff bases also exhibited three mass loss steps (Figure 3). Initially, the complexes underwent a dehydration process, followed by the decomposition which occurred in two stages; the final stage was related to the burning of decomposed material, leading to the formation of residue after the decomposition.

Table 3 shows values of mass loss percentages at each step, temperature ranges, percentages of residue generated after burning of the material and the peak temperature in the DTA curves. According to these data, it is possible to suggest that when the polymers interacted more effectively with the metal, the highest percentages of residue were obtained after burning. The results of thermogravimetric analyses demonstrated that the H–Ch, MeO–Ch and NO_2_–Ch ligands have lower thermal stability than their complexes with palladium(II) and platinum(II). By comparing the values of the calculated amount of theoretical residue with the actual amount of residue after the experiment, it is possible to conclude that the reactions with platinum had a higher yield of complexation than those with palladium.

Schiff base complexes of palladium Pd–H–Ch and platinum Pt–H–Ch were thermally decomposed at 1000 °C and the complexes resulted in a black residue. These residues’ X-ray powder diffraction analysis patterns were compared with MIDI JADE 5.0 software in order to identify the diffraction pattern. For the sample Pd–H–Ch, some peaks were observed in the 2θ range of 10°–80°, and the major peaks were indexed as 34°, 40°, 42°, 45°, 55°, 60°, 68° and 71°. These peaks are identical to those the indexes from the PdO crystal structure. For Pt–H–Ch, the major peak indexes were 40°, 46°, 68°, 81°, 86°, 103° and 118°, which are identical to the index of the crystal structure for PtO [31]. Through this analysis, it was possible to confirm the formation of PdO and PtO as the final products of decomposition. The values (%) of residue were 8.0%, 10.8%, 9.6% and 15.8% for palladium compounds Pd–Ch, Pd–H–Ch, Pd–MeO–Ch and Pd–NO_2_–Ch, respectively. For the platinum complexes, the percentages of residue were 8.5%, 20.2%, 20.3% and 20.2% for Pt–Ch, Pt–H–Ch, Pt–MeO–Ch and Pt–NO_2_–Ch, respectively (shown in Table 3). The calculated amounts of theoretical residue were determined by assuming that the complexation occurs in all units replaced with Schiff bases. Comparing these values with those obtained by the percentage of experimental residue, it was verified that metal ions interact more with Schiff base type ligands than with chitosan. Overall, compared to the other ligands, the NO_2_–Ch ligand (5-nitro-salicylaldehyde) demonstrated higher metal-ligand interactions, but the obtained residue percentages indicate that its interaction with platinum was more efficient than with palladium.

### 2.4. X-ray Powder Diffraction Spectroscopy (XPRD)

Compared to other naturally occurring carbohydrate polymers, chitosan exhibits relatively higher crystalline character because of its strong intermolecular hydrogen bonding [32]. The XRD spectra of Ch, H–Ch and the Pd–H–Ch, Pt–H–Ch complexes are given in Figure S3 (available in supplementary material). Chitosan has characteristic peaks at 2θ = 20° in the XRD spectrum, in accordance with a previous report [33]. Crystallinity indexes of Ch, H–Ch and the biomaterial complexes with Pd and Pt were determined using Equation (2):

(2) $$\mathrm{Crystallinity}\;\mathrm{Index}\;\left(\%\right)=\frac{I_{110}-I_{\mathrm{am}}}{I_{110}}\times 100$$

In Equation (2), $I_{110}$ is the maximum intensity at 2θ in 20° and $I_{\mathrm{am}}$ is the intensity of the amorphous portion diffraction at 2θ ~ 16°. When chitosan was chemically modified with salicylaldehyde and then complexed with Pd(II) and Pt(II), a decrease in the crystallinity index was observed. The order of the crystallinity index (%) of the compounds was:

CCh (62.3) ~ Ch (61.9) > Pd–H–Ch ~ Pt–H–Ch.

This decrease in the crystallinity index confirms the formation of the Schiff bases; and, the widening of peaks as well as the decreases in intensity observed in the XRD diagram of all complexes demonstrate that the complexes have a less crystalline character than the ligands.

### 2.5. Scanning Electron Microscopy (SEM) with X-ray Energy-Dispersive Analysis (EDS)

**Figure 4:**
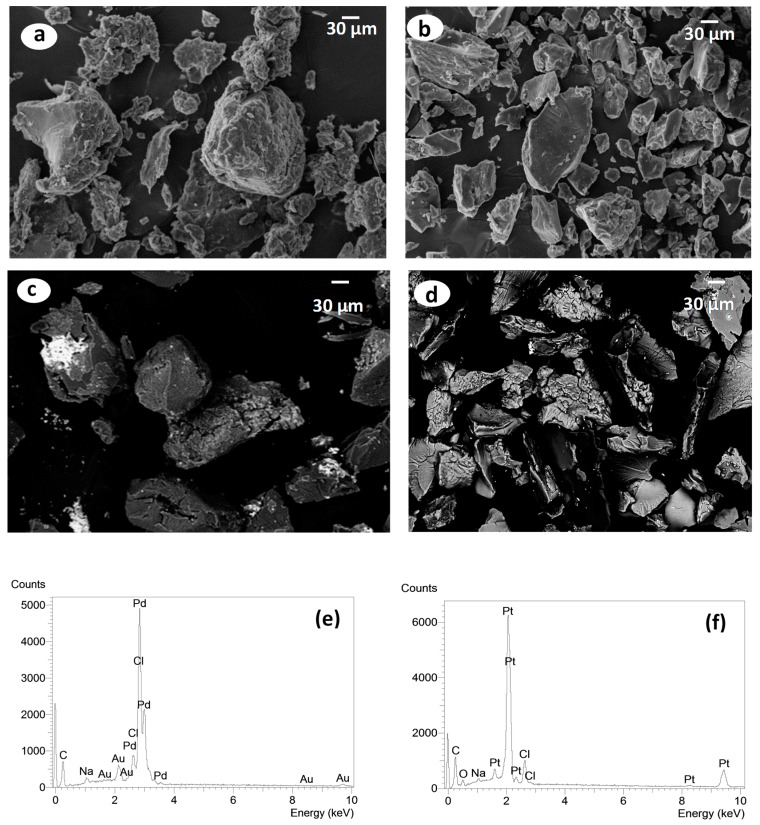
Scanning electron microscopy (SEM) pictures of (**a**) Ch; (**b**) H–Ch; (**c**) Pd–H–Ch; (**d**) Pt–H–Ch and X-ray energy-dispersive analysis (EDS) spectra of bright spots (**e**) Pd–H–Ch and (**f**) Pt–H–Ch.

Scanning electron microscopy (SEM) can reveal the morphology of solid compounds. In the micrographs presented in Figure 4a–d, for Ch, H–Ch, Pd–H–Ch and Pt–H–Ch, respectively, the compounds are seen to have granular shapes and irregular sizes with smooth and rough surface regions.

In SEM images, the energy dispersive detector was used for imaging the complexes to observe the brighter regions that represented the metallic species, confirming the presence of Pd(II) and Pt(II) ions. The spectra of X-ray energy dispersive analysis on the bright spots seen in SEM micrographs of the Pd–H–Ch and Pt–H–Ch complexes are shown in Figure 4e,f, respectively. In Figure 4e, the percentages of chlorine and palladium in the bright spots were 3.8% and 81.1% (*m*/*m*), whereas in Figure 4f, the percentages of chlorine and platinum in the bright spots were 4.5% and 60.1% (*m*/*m*), respectively. In the darker areas of the same Pd–H–Ch sample, the percentages of chlorine and palladium were 7.2% and 23.3% (*m*/*m*), respectively, whereas the darker areas of the Pt–H–Ch sample had percentages of chlorine and platinum that were 13.3% and 25.1% (*m*/*m*), respectively (Figure S4 available in supplementary material). These results demonstrate that metallic ions interacted efficiently with Schiff bases. Traces of gold and sodium can arise from the preparation of the sample for the measurement and purification processes, respectively.

### 2.6. Electronic Absorption Spectra

The electronic transitions in metallic complexes, such as in polymers, occur due to the presence of chromophore groups present along the polymer chain. Therefore, if the chromophore groups have π electrons, an absorption band appears in the visible and UV region [19]. The electronic spectra of Schiff base ligands CC, Ch, MeO–Ch, NO_2_–Ch, as well as palladium(II) and platinum(II) complexes were recorded in the 200–800 nm range in the UV-Vis region for solid samples. In addition, diffuse reflectance measurements were performed, applying the Kubelka–Munk Equation [34]. The electronic spectra of ligands show two important absorption bands before the 460 nm region. These bands are attributed to the π→π* and n→π* transitions of azomethine groups C=N. Bands attributed to the π→π* transitions of C=C observed in aromatic rings are not clearly defined, probably because the spectra were obtained from solid samples.

The bands of C=N attributed to π→π* transitions are observed in the spectra of all the complexes, with small shifts in wavelength either to lower or higher energies. These shifts represent the chemical modification of ligands with the metal ions. Another significant piece of evidence for coordination is the appearance of a band in the electronic spectra for palladium and platinum complexes around 500 nm, which is a characteristic absorption band related to the *d–d* electronic transition. Because the geometry of the complexes is attained from the λ max values of these *d-d* transitions, and the electronic spectra of palladium and platinum show bands around 460 and 500 nm, respectively, one can infer the square planar geometry of the complexes [35]. 

The electronic spectra of diamagnetic palladium complexes show bands at 360–495 nm for Pd–H–Ch, 370–475 nm for Pd–MeO–Ch and 307–505 nm for Pd–NO_2_–Ch. These bands are related to transitions in a square planar configuration [36]. Accordingly, the same behavior was observed for platinum complexes; the electronic spectra of these complexes showed bands at 361–475 nm for Pt–H–Ch, 358–470 nm for Pt–MeO–Ch and 315–432 nm for Pt–NO_2_–Ch, also suggesting a square planar configuration for these complexes.

### 2.7. Antimicrobial Activity

The antimicrobial effects of chitosan and its derivatives were assessed separately in vitro against two economically important plant pathogens, the Gram-negative bacterium *Pseudomonas syringae* pv. *tomato*, the causal agent of bacterial speck of tomato, and the cereal head blight fungus *Fusarium graminearum*.

Results of the antibacterial and antifungal assays are summarized in Table 4. Percentage growth was calculated and plotted with different concentrations tested to obtain the concentration that inhibited 50% of growth (IC_50_). The minimum inhibitory concentration (MIC) was defined as the lowest concentration that fully inhibited microbial growth after 24 h.

The obtained data indicate that chitosan and its derivatives had significant antibacterial and antifungal effects. It was also observed that antimicrobial activity increased with increasing concentrations of chitosan and its derivatives.

In the antibacterial assay, results showed that the chemical modification of chitosan enhanced its antibacterial activity: all Schiff bases H–Ch, MeO–Ch and NO_2_–Ch and their complexes were more active than the nonmodified chitosan in inhibiting the growth of *P. syringae*, with IC_50_ ranging from 7–15 μg mL^−1^ for the modified chitosan compounds and 42 μg mL^−1^ for the nonmodified chitosan (Table 4). The most active compounds were the salicylaldehyde chitosan H–Ch and its complexes Pt-H–Ch and Pd–H–Ch (MIC of 25 μg mL^−1^), while the other Schiff bases and their complexes were less active (MIC higher than 50 μg mL^−1^). When comparing Pd(II) and Pt(II) complexes to the Schiff bases, these tests showed that both Schiff bases and their complexes have similar antibacterial effects (Figure S5 available in supplementary material).

The antibacterial effects of chitosan and its derivatives have been reported by many authors [9,12,21,23,37,38]. Although several mechanisms for their antibacterial activity have been suggested, the exact mode of action is still not known in detail. However, there is clear evidence of these compounds having molecular-level interactions with the cell membrane [9,39]. Ionic and/or hydrophobic interactions are often considered to be common interactions that lead to the damage or breakage of cell membranes [40,41]. Results of the antibacterial assay showed that the hydrophobic chitosan Schiff bases had stronger antibacterial effects than the nonmodified chitosan; moreover, large effects were observed for the more hydrophobic derivatives H–Ch, MeO–Ch, NO_2_–Ch, respectively. These results confirm the importance of hydrophobic interactions between chitosan derivatives and the cell membrane reported in previous studies. In fact, the importance of these nonelectrostatic types of interactions has been demonstrated at the molecular level in studies with model membranes. For instance, Pavinatto et al. [41], showed that O-acyl chitosan derivatives caused large expansions and changes in the elasticity of the model membrane and could be incorporated into the hydrophobic region of the phospholipids even at high surface pressures.

In the antifungal assay, the modification of chitosan decreased its antifungal activity, where Schiff bases had a MIC ranging from 40 to 60 μg mL^−1^ compared to 30 μg mL^−1^ for the nonmodified chitosan (Table 4). The antifungal activities of Schiff bases decreased in the order of Ch > NO_2_–Ch > H–Ch > MeO–Ch; the Pd(II) and Pt(II) complexes were least active, with a MIC of 60 μg mL^−1^ or higher. This may be explained by the difference in the degree of substitution ($\bar{\mathrm{DS}})$ of Schiff bases: as ($\bar{\mathrm{DS}})$ increases for NO_2_–Ch > H–Ch> MeO–Ch, the antifungal effect decreases in the same order. In this case, the greater number of protonated amine groups, the higher the antimicrobial activity. In contrast to the results of the antibacterial assay, here the hydrophobic interactions appear to be less crucial for the action of chitosan and its derivatives against *F. graminearum* (Figure S6 available in supplementary material). Thus, charge density seems to be an important factor for the antifungal effect, where chitosan Schiff bases with fewer available amine groups were less active than the nonmodified chitosan, and the major mode of action is the electrostatic interaction between the positive charge of chitosan and the cell surface of the fungi [9]

The difference in effects observed in the antifungal and antibacterial assays suggests that the tested Schiff bases act by different mechanisms depending on the tested microorganisms, such that hydrophobic interactions seem to be more crucial for activity against *P. syringae*, but electrostatic interactions seem to be more important for activity against *F. graminearum*. It is interesting to note that the composition of the microorganisms’ cell membranes has been shown to play an essential role in the action of chitosan; for example, Verlee et al. [9] suggested that depending on their cell membrane structure, microorganisms can be divided into four groups: Gram-positive bacteria, Gram-negative bacteria, chitosan-sensitive fungi and chitosan-resistant fungi [42]. However, little is known about the interaction between chitosan derivatives and the different cell membranes.

In our study, we showed for the first time that chitosan Schiff bases with different hydrophobic groups act differently on fungi and bacteria. However, studies on different microorganisms or different model membranes need to be conducted to generalize this hypothesis. More importantly, both the ratio between free amino groups and substituted amino groups and the balance between the hydrophilicity and hydrophobicity of the chitosan derivatives need to be optimized depending on the desired microorganism.

### 2.8. Antitumor Assay

**Figure 5:**
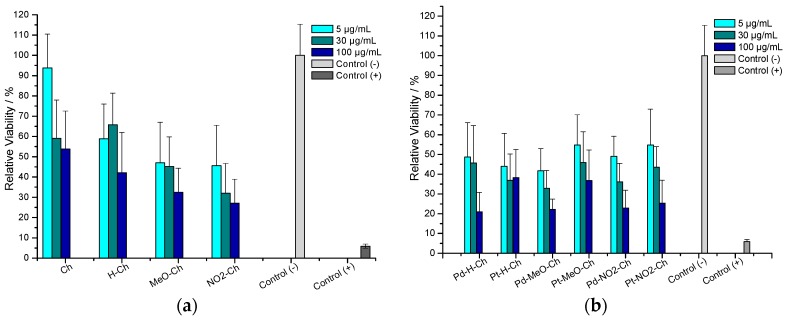
Cytotoxicity of chitosan, Schiff bases and Pd(II) and Pt(II) complexes against MCF-7 cells in 96-well plates determined using the MTT assay. (**a**) Salicylaldehyde Schiff bases and (**b**) Pd(II) and Pt(II) complexes at concentrations of 5, 30, and 100 μg mL^−1^. Measurements were conducted for 24 h. Eight replicates were performed for each treatment, and the experiment was carried out three times, from which the standard deviation was calculated.

The in vitro cytotoxicity effects of chitosan, Schiff bases and Pd(II), Pt(II) complexes were evaluated against breast cancer cells using the MTT assay. Results showed that for all the test compounds, the relative viability of the cells decreased with an increase in the concentration of the compound. In Figure 5a, chitosan Schiff bases showed higher antitumor activity, with cell viability ranging from 30% to 70%, compared to nonmodified chitosan, which showed a cell viability higher than 70% at all tested concentrations. Moreover, it was observed that the percentage of cell viability decreased (higher to lower cell viability) in the order of Ch > H–Ch > MeO–Ch > NO_2_–Ch. Results of the MTT assay for Pd(II) and Pt(II) complexes revealed a slight decrease in the viability of MCF-7 cells after 24 h compared to cells treated with the free Schiff bases (Figure 5b). One explanation could be that the square planar geometry of the Pd(II) and Pt(II) complexes allows them to interact with the secondary structure of DNA, potentially even inhibiting the replication of DNA [43]. Moreover, it has been reported that the antitumor activity of such complexes probably involves the inhibition of ribonucleotide reductase, which converts ribonucleotides to deoxyribonucleotides, leading to changes in genetic material [44]. 

In fact, the platinum complex cisplatin has been employed as an important drug in chemotherapy medications [45]. However, its effectiveness is limited due to its high toxicity [46]. In this study, we showed that the platinum and palladium complexes prepared from chitosan Schiff bases had strong antitumor effects and could potentially be used as a safer alternative in chemotherapy.

## 3. Materials and Methods

### 3.1. Materials

Commercial chitosan of low molecular weight ($\bar{\mathrm{Mw}}$) 334.3 kDa and high degree of deacetylation $\left(\bar{\mathrm{DD}}\right)$ 90.1% was purchased from Sigma-Aldrich, St. Louis, MO, USA. Palladium(II) chloride (P.A) was obtained from Merck (Kenilworth, NJ, USA). Platinum salt was obtained from reaction of solid platinum. One g of solid platinum was dissolved in hydrochloric and nitric acid (3:1, *v*/*v*) for 24 h (8 h/day). The solution was heated in a beaker and capped with a flask filled with water to avoid evaporation of the solution, and after 4 h, 1 mL of nitric acid was added. On the fourth day, the chloroplatinic acid solution was evaporated in a covered beaker with an empty round bottom flask, just to avoid loss of the material during evaporation. This concentrated solution was cooled to room temperature and a volume of hydrochloric acid was added to the mixture. Then the solution was heated, and the release of a brown gas was observed, indicating the presence of nitric oxides. To assure that all HNO_3_ was effectively eliminated, concentrated HCl was added again and the material was evaporated until the absence of the brown fumes. The product was kept in a desiccator under reduced pressure. This product was used as platinum source for the reaction with the Schiff bases. Benzaldehydes: Salicylaldehyde, 5-methoxy-salicylaldehyde, and 5-nitro-salicylaldehyde were obtained from Sigma-Aldrich (98% purity), deuterium oxide and dimethyl sulfoxide (DMSO) were purchased from Sigma-Aldrich, USA. 3-(4,5-dimethylthiazol-2-yl)-2,5-diphenyltetrrazolium bromide (MTT) and RPMI-1640 cell culture medium were purchased from Sigma-Aldrich GmbH, Mannheim, Germany. Ultrapure MilliQ water was used throughout. Autoclaved, double deionized, ultrapure water (ddH_2_O, MilliQ 18.2 MΩ/cm) was used for the microbiological experiments.

### 3.2. Chitosan Purification

Chitosan purification was attained by the dissolution of 15 g in 4.5 L of 0.50 mol L^−1^ acetic acid solution, overnight under agitation, followed by precipitation in the hydrogel form by carefully adding concentrated NH_4_OH. Chitosan was washed with water until neutrality, followed by ethanol washing. The final product was dried at 40 °C under low pressure and kept in a desiccator until further use [47].

### 3.3. Synthesis of Biopolymeric Schiff Bases

Salicylaldehyde and its 5-methoxy and 5-nitro derivatives were used for the synthesis of Schiff bases by dissolving 6 g of previously purified chitosan, dissolved in 0.10 mol L^−1^ acetic acid, and stirred at room temperature overnight. Then, the desired salicylaldehyde derivative, previously dissolved in 20 mL of ethanol, was added dropwise to the chitosan solution. The amounts of chitosan and salicylaldehyde followed the molar ratio of 1.5 salicylaldehyde/1.0 chitosan (mol/mol). This mixture was kept in a homemade reactor vessel immersed in a thermostated bath at 55 °C and stirred for 18 h to assure its dissolution in a hydrogel form. The preparation of the Schiff base was achieved with formation of yellow gels. The products were collected by filtration, washed several times with water and ethanol to remove any unreacted aldehyde, dried at 40 °C and kept in a desiccator over silica gel. Under these conditions, enhanced substitution can be achieved [48].

### 3.4. Synthesis of Pd(II) and Pt(II) Complexes

Preparation of Pd(II) complexes was performed such that a solution of PdCl_2_ (1 mmol) in 10 mL of ethanol was added to the ligand solution (1 mmol in 20 mL of ethanol) while stirring. The solution was maintained at 40 °C for 8 h in the presence of four drops of concentrated hydrochloric acid. The same procedure was used in preparation of Pt(II) complexes [29] .

### 3.5. Characterization

#### 3.5.1. ^1^H-NMR Spectroscopy

The ^1^H nuclear magnetic resonance (^1^H-NMR) spectra were obtained using an Agilent Technologies, Santa Clara, CA, USA. Model 400/54 Premium Shielded spectrometer. For these measurements, a suspension of 10.0 mg of chitosan with 1.0 mL of 1% HCl/D_2_O (*v*/*v*) was stirred for 18 h. All the measurements were performed at 70 °C.

#### 3.5.2. Size Exclusion Chromatography

The weight average molecular weight (M_w_), the number average molecular weight (M_n_) and the index of polydispersity (I_p_) of commercial chitosan CCh and purified chitosan Ch were measured by size exclusion chromatography (SEC-MALS-RI) using an Agilent 1200 Series with isocratic pump and Novema^®^ (Polymer Standards Service GmbH, Mainz, Germany) columns coupled online with a refractive index detector (Agilent Series 1200) RID and multi-angle laser light scattering (PSS SLD 7000 MALLS^®^ (Brookhaven Instruments, New York, NY, USA) equipped with a 5 mW He/He laser operating at λ = 632.8 nm. The degassed mobile phase consisted of ammonium acetate 0.2 mol L^−1^/acetic acid 0.15 mol L^−1^, pH 4.5 and the flow rate was 0.7 mL/min (35 °C). Data were evaluated using the software WinGPC 7.0.1 (Polymer Standards Service GmbH, Mainz, Germany).

#### 3.5.3. Infrared Spectroscopy

FTIR absorption spectra of chitosan and ligands were obtained in an IRAffinity-1 FTIR spectrophotometer (Shimadzu, Quito, Japan) operated in the range of 4000–400 cm^−1^, with resolution of 4 cm^−1^ and accumulation of 32 scans. The samples were prepared in the form of pellets with KBr (sample/KBr = 1% *w*/*w*) and a pure KBr disk was used as a reference. Infrared absorption spectra of the complexes were recorded in an MB 102 FTIR spectrophotometer (ABB-Bomen-Michelson, Ville of Quebec, QC, Canada) in the range of 4000–200 cm^−1^. The polysaccharide sample (10 mg) was dried overnight at 40 °C under reduced pressure and mechanically well homogenized with 100 mg of CsI in agate mortar and pressed. The preparation of the pellets was performed under the light of an incandescent lamp (60 W) and IR spectra were recorded against a CsI disk as a blank by accumulation of at least 64 scans with a resolution of 2 cm^−1^.

#### 3.5.4. Thermal Analysis

Thermogravimetry (TGA), derivative thermogravimetry (DTG) and differential thermal analysis (DTA) were performed in an SDT-Q600 modulus from TA Instruments. The TG/DTG-DTA curves of the chitosan and biopolymeric Schiff bases were obtained under a dry air atmosphere (100 mL min^−1^), open α-alumina sample holder, sample mass around 6 ± 1 mg and heating rate of 10 °C min^−1^ from 25 °C to 1000 °C, under room pressure. The parameters were calculated using the Thermal Advantage^®^ Software (v.2.5.0.256) (TA Instruments, New Castle, DE, USA) and the modulus was calibrated according to the manufacturer’s instructions for mass and temperature.

#### 3.5.5. X-ray Powder Diffraction (XPRD)

X-ray powder diffraction patterns were obtained using a Rigaku Ultima IV (Rigaku, Tokyo, Japan) X-ray diffractometer to evaluate residues of thermal decomposition and the crystallinity of the ligand and complexes using Cu Kα radiation (λ = 15,406 nm) with a generator at 40 kV and 30 mA at 25 °C. The diffraction angle (2θ) range for XRD spectroscopy from 0° to 100° was attained with a rate of 2° min^−1^.

#### 3.5.6. Scanning Electron Microscopy (SEM) and Energy Dispersive X-ray (EDAX)

Photomicrographs of the polymers and the complex were obtained using scanning electron microscopy (SEM, Cambridge, UK) with energy-dispersive X-ray analysis (EDAX, Cambridge, UK). Observation was performed using a Zeiss LEO 440 (Cambridge, UK) with Oxford detector (model 7060) scanning electron microscope operating at 15 kV electron beam, equipped with an EDAX Link Analytical (Isis System Series 300, Oxford, UK) with detector from SiLi Pentafet ATW II (Atmosphere Thin Window, Oxford, UK), resolution of 133 eV to 5.9 eV and 10 mm^2^ area. For the calibration, a standard of Co of 20 kV electron beam was used, focal length 25 mm, dead time of 30%, current of 2.82 A and I probe of 2.5 nA.

#### 3.5.7. Electronic Absorption Spectra

Electronic absorption spectral analysis was carried out using a Shimadzu UV-3600 spectrophotometer in the wavelength range of 200–800 nm. All spectra were obtained using solid samples previously dried in an oven at 40 °C. Approximately 2 mg of sample was dispersed in 20 mg of barium sulfate, which was also previously dried.

#### 3.5.8. Antibacterial Assay

Chitosan, Schiff bases and their complexes were tested in vitro for their antibacterial activity against *Pseudomonas syringae* pv. *tomato*. The strain was provided by Leibniz—Institut DSMZ (Braunschweig, Germany). Bacteria were initially grown in NYG medium (0.5% (*w*/*v*) peptone, 0.3% (*w*/*v*) yeast extract and 2% (*v*/*v*) glycerol) at 30 °C under agitation at 100 rpm for two days. The antibacterial assay was conducted in a 96-well microtiter plate by mixing 40 μL of chitosan or its derivatives with 160 μL of bacterial suspension OD = 0.0125 or media for the blank. Bacterial growth was measured during 24 h at 5 min intervals at 26 °C. The absorbance was measured at λ = 600 nm in a microplate reader using a UV/Vis-spectrophotometer (Molecular Devices, SpectraMax M2, Sunnyvale, CA, USA). Four independent experiments were carried out, each in triplicate, and the data are reported as means ± SD.

#### 3.5.9. Antifungal Assay

The antifungal activity of chitosan, Schiff bases and the complexes was tested against the cereal pathogen *Fusarium graminearum* using a 96-well microtiter plate. The fungus was isolated in Wuhan (China) and the assay was performed according to Oliveira et al. [49]. Aliquots (150 μL) of sterile complete medium (CM), pH 4.3, were dispensed into the wells with 40 μL of chitosan, Schiff base or complexes; water was used as a control. For a dose response relationship, treatments were used at the following concentrations: 20, 30, 40, 50 and 60 μg mL^−1^. Finally, 10 μL of a spore suspension of *F. graminearum* (7 × 10^4^ sp mL^−1^) or 10 μL of sterile water (blanks) were added to the mixture. The plates were incubated at 26 °C under agitation, 200 rpm, for five days. Fungal growth was assessed by measuring the optical density of the culture media at 600 nm using a UV/Vis-spectrophotometer (Thermo Fisher Scientific Multiscan GO 60, Waltham, MA, USA) every 24 h. Three independent experiments were carried out, each in triplicate, and the data are reported as means ± SD.

#### 3.5.10. In Vitro Cytotoxicity Assay

The cytotoxicity of chitosan, Schiff bases and the complexes was evaluated using the MTT assay [50]. Initially, 100 μL of MCF-7 breast cancer cell suspension was transferred to a 96-well tissue culture plate (~10^4^ cells per well or ~10^5^ cells mL^−1^) and allowed to attach for 24 h. Cells were washed with PBS buffer then samples were added and plates were incubated for another 24 h.

Afterwards, the samples were removed and replaced by 100 μL of medium, and 25 μL of MTT solution in PBS (5 mg mL^−1^) were added to each well. After 3 h of incubation, the medium was removed and the dye was dissolved in DMSO. Plates were shaken at 300 rpm for 10 min and the absorbance was measured at λ = 570 nm in a microplate reader using a UV/Vis-spectrophotometer (Thermo Fisher Scientific Multiscan GO 60, Waltham, MA, USA). Relative viability values were calculated by dividing individual viability data by the mean of the negative control (untreated cells). As a positive control, we used 4% Triton X-100 in PBS (Sigma Aldrich, St. Louis, MO, USA).

## 4. Conclusions

Chitosan with salicylaldehyde, 5-methoxy and 5-nitro salicylaldehyde, and their palladium(II) and platinum(II) complexes derived from condensation were synthesized and characterized by different spectroscopic and thermal analytical techniques. Our study showed that chitosan derivatives of Schiff bases can be used for the complexation of palladium and platinum ions. The percentages of residue observed by thermal analysis were satisfactory for palladium and platinum complexes; however, the interaction with platinum was higher than with palladium. Results of FTIR revealed that the Schiff base ligands are probably coordinated to the central metal ion through nitrogen and oxygen. Complex formation was primarily evidenced by SEM and EDS results, and large differences in the percentage of metals were observed in different regions of the samples.

In general, TGA showed that the complexes are thermally less stable than the ligands, and evaluation of the powder X-ray diffraction indicated that the complexes were less crystalline than the chitosan and the Schiff bases. Electronic spectra also confirmed the complexation by the presence of bands at 430–505 nm related to transitions *(d-d)* that suggest a square planar geometry of the complexes. More interestingly, all chitosan derivatives exhibited antimicrobial and antitumor activity at very low concentrations. Regarding antimicrobial assay, chitosan Schiff bases were more active against the bacterium *Pseudomonas syringae* pv. *tomato* than against the fungus *Fusarium graminearum*, which confirms that chitosan and its derivatives have different modes of action depending on the tested microorganism. However, no considerable differences were observed between Schiff bases and their complexes in the same assay. Moreover, the free Schiff bases and their palladium(II) and platinum(II) complexes exhibited high antitumor activity against the breast cancer cell line MCF-7.

## Figures and Tables

**Table 1:** Results of ($\bar{\mathrm{DD}}$), $\left(\bar{\mathrm{DS}}\right)$, ($\bar{\mathrm{Mw}}$), and ($\bar{\mathrm{MM}}$) for chitosans and Schiff bases.

Polymers	$\bar{DD}$/%	$\bar{DS}$/%	$\bar{Mw}$/kDa	$\bar{MM}$/g mol^−1^
CCh	90.1	-	334.3	165.4
Ch	90.4	-	222.9	165.3
H-Ch	-	51.2	294.7 *	218.2
MeO-Ch	-	54.3	321.0 *	237.7
NO_2_-Ch	-	48.0	319.3 *	236.4

* ($\bar{\mathrm{Mw}}$) calculated using the values of ($\bar{\mathrm{MM}}$) and DP = Degree of polymerization.

**Table 2:** Data obtained from TGA/DTG and DTA curves of CCh, Ch, MeO–Ch and NO_2_–Ch.

Sample	Process (Air Atmosphere)	TGA/DTG			DTA
		Temperature Range/°C	Mass Loss/%	^a^ Ratio	Temperature Peak/°C *
CCh	Dehydration	22.2–171.5	7.2		71.3 (endo)
	1st step	171.5–382.3	51.7	1.27	303.9 (exo)
	2nd step	382.3–707.5	40.6		481.6 (exo)
Ch	Dehydration	22.3–182.9	8.1		76.9 (endo)
	1st step	182.9–402.2	47.5	1.07	303.9 (exo)
	2nd step	402.2–713.2	44.5		541.1 (exo)
H-Ch	Dehydration	22.2–128.0	3.8		60.9 (endo)
	1st step	128.0–390.8	41.5	0.76	313.3 (exo)
	2nd step	390.8–713.2	54.6		543.0 (exo)
MeO-Ch	Dehydration	23.3–107.2	2.4		188.5 (endo)
	1st step	107.2–358.7	43.6	0.80	313.3 (exo)
	2nd step	358.7–727.3	54.3		500.5 (exo)
NO_2_-Ch	Dehydration	22.2–146.0	3.2		78.9 (endo)
	1st step	146.0–368.1	39.6	0.69	312.4 (exo)
	2nd step	368.1–733.0	57.1		496.7 (exo)

* (endo = endothermic; exo = exothermic), ^a^ Ratio between the mass losses in the first and second step (first/second), after water loss.

**Table 3:** Data obtained from TGA/DTG and DTA curves of palladium and platinum complexes.

Sample	Process (Air Atmosphere)	TGA/DTG				DTA
		^a^ ΔT/°C	Mass Loss/%	^b^ Residue Exp/%	^c^ Residue Calc/%	Peaks/°C *
Pd-Ch	Pd-Ch·nH_2_O → Pd-Ch + nH_2_O	24.1–181.9	12.0	-	-	65.6 (endo)
	Pd-Ch → Res-Pd	181.9–357.7	43.5	-	-	221.6 (endo)
	decomposition of Res-Pd	357.7–648.9	35.2	-	-	426.8 (exo)
	residue PdO	-	-	8.0	40.7	820.0 (endo)
Pd-H-Ch	Pd-H-Ch·nH_2_O → Pd-H-Ch + nH_2_O	23.1–172.4	8.7	-	-	58.1 (endo)
	Pd-H-Ch → Res-Pd	172.4–367.5	39.2	-	-	224.1 (endo)
	decomposition of Res-Pd	367.5–560.7	36.6	-	-	426.8 (exo)
	residue PdO	-	-	10.8	19.3	809.4 (endo)
Pd-MeO-Ch	Pd-MeO-Ch·nH_2_O → Pd-MeO-Ch + nH_2_O	23.6–165.8	7.5	-	-	73.8 (endo)
	Pd-MeO-Ch → Res-Pd	165.8–307.1	32.8	-	-	236.0 (exo)
	decomposition of Res-Pd	307.1–626.6	48.9	-	-	361.1 (exo)
	residue PdO	-	-	9.6	19.3	813.3 (endo)
Pd-NO_2_-Ch	Pd-NO_2_-Ch·nH_2_O → Pd-NO_2_-Ch + nH_2_O	24.1–173.4	10.5	-	-	73.2 (endo)
	Pd-NO_2_-Ch → Res-Pd	173.4–352.1	38.3	-	-	233.9 (exo)
	decomposition of Res-Pd	352.1–631.9	34.3	-	-	399.3 (exo)
	residue PdO	-	-	15.8	17.1	813.4 (endo)
Pt-Ch	Pt-Ch·nH_2_O → Pt-Ch + nH_2_O	20.8–144.0	7.2	-	-	61.5 (endo)
	Pt-Ch → Res-Pt	144.0–360.2	42.7	-	-	239.8 (endo)
	decomposition of Res-Pd	360.21–720.4	41.9	-	-	415.2 (exo)
	residue PdO	-	-	8.5	53.0	512.9 (exo)
Pt-H-Ch	Pt-H-Ch·nH_2_O → Pt-H-Ch + nH_2_O	19.8–167.7	11.3	-	-	58.1 (endo)
	Pt-H-Ch → Res-Pt	167.7–319.4	31.0	-	-	234.6 (endo)
	decomposition of Res-Pt	319.4–583.0	36.5	-	-	444.0 (exo)
	residue PtO	-	-	20.2	26.2	-
Pt-MeO-Ch	Pt-MeO-Ch·nH_2_O → Pt-MeO-Ch + nH_2_O	19.8–163.9	7.2	-	-	68.5 (endo)
	Pt-MeO-Ch → Res-Pt	163.9–371.5	38.2	-	-	239.6 (endo)
	decomposition of Res-Pt	371.5–587.4	34.5	-	-	426.7 (exo)
	residue PtO	-	-	20.3	26.5	-
Pt-NO_2_-Ch	Pd-NO_2_-Ch·nH_2_O → Pd-NO_2_-Ch + nH_2_O	21.3–157.3	8.9	-	-	-
	Pd-NO_2_-Ch → Res-Pd	157.3–350.7	36.5	-	-	226.9 (endo)
	decomposition of Res-Pd	350.7–539.3	34.5	-	-	444.0 (exo)
	residue PdO	-	-	20.2	23.5	-

^a^ = temperature range; ^b^ = experimental residue; ^c^ = calculated residue assuming that the complexation occurs in all units replaced with Schiff bases; Res = residue; * (endo = endothermic; exo = exothermic).

**Table 4:** Antibacterial and antifungal activity of chitosan, Schiff bases and Pd(II), Pt(II) complexes against *P. syringae* and *F. graminearum*.

	*P. syringae*		*F. graminearum*	
Sample	MIC μg mL^−1^	IC_50_ μg mL^−1^	MIC μg mL^−1^	IC_50_ μg mL^−1^
Chitosan Schiff bases				
Ch	>50	42	30	24
H–Ch	25	13	50	44
MeO–Ch	>50	14	60	54
NO_2_–Ch	>50	9	40	34
Complexes				
Pd–H–Ch	25	7	60	55
Pd–MeO–Ch	>50	14	>60	56
Pd–NO_2_–Ch	>50	14	>60	>60
Pt–H–Ch	25	10	60	55
Pt–MeO–Ch	>50	15	>60	>60
Pt–NO_2_–Ch	>50	15	60	53

## Supplementary Materials

Supplementary materials are available online.
