# Supplementary material for: Synthesis, Characterization and Biological Activities of Biopolymeric Schiff Bases Prepared with Chitosan and Salicylaldehydes and Their Pd(II) and Pt(II) Complexes
Source: Molecules. 2017 Nov 16;22(11):1987. doi: 10.3390/molecules22111987 (PMC6150178; doi:10.3390/molecules22111987)
Supplement: Supplementary file 1 [file molecules-22-01987-s001.pdf]

Supplementary Material

Synthesis, characterization and biological activities of biopolymeric Schiff bases prepared with chitosan and salicylaldehydes and their Pd(II) and Pt(II) complexes

Hellen F. G. Barbosa [1] Maha Attjioui [3], Ana Paula G. Ferreira [1], Edward R. Dockal [2], Nou Eddine El Gueddari[3,] Bruno M. Moerschbacher [3,]*, Éder T. G. Cavalheiro [1,]*

[1] *Instituto de Química de São Carlos, Universidade de São Paulo, Av. Trabalhador São Carlense, 400,13566-590 São Carlos, SP, Brazil*

[2] *Universidade Federal de São Carlos, Via Washington Luis, Km 235, 13560-900, São Carlos, SP, Brazil*

[3] *Institute of Plant Biology and Biotechnology (IBBP), Westfälische Wilhelms-Universität Münster, Schlossplatz 8, 48143 Münster, Germany*

*Correspondence: moersch@uni-muenster.de; phone: +49 251 83-24794

*Correspondence: cavalheiro@iqsc.usp.br; phone: +55-16-3373-8054

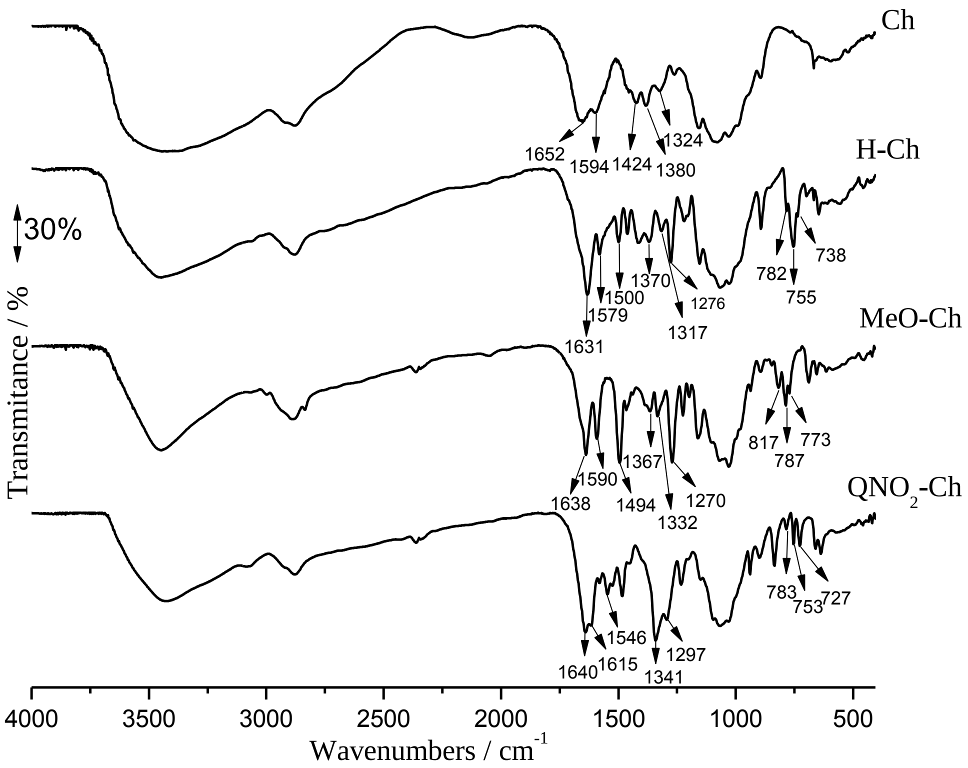

Figure S1. FTIR of chitosan and Schiff bases

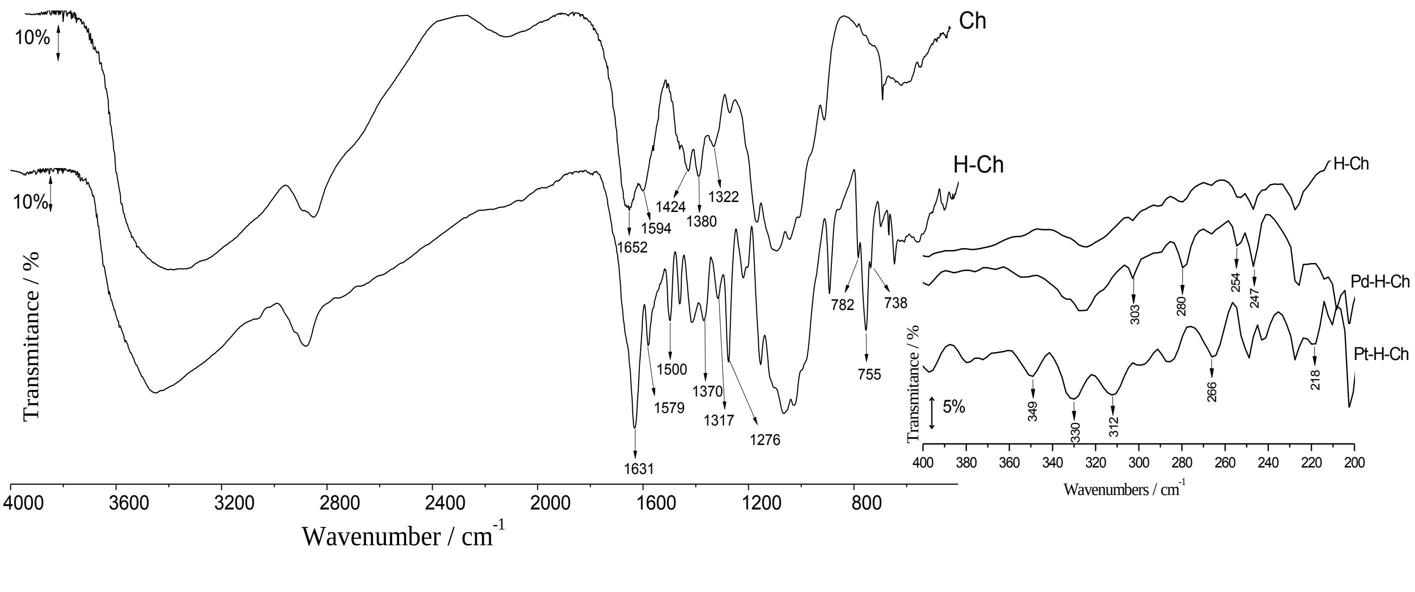

Figure S2. FTIR of Ch, H-Ch compared with Pd-H-Ch and Pt-H-Ch.

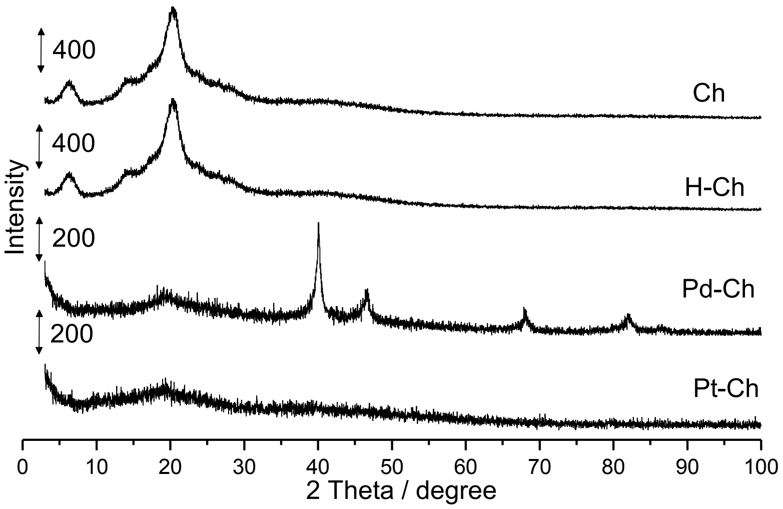

Figure S3.XRD of Ch, H-Ch biopolymers and Pd-Ch, Pt-Ch complex.

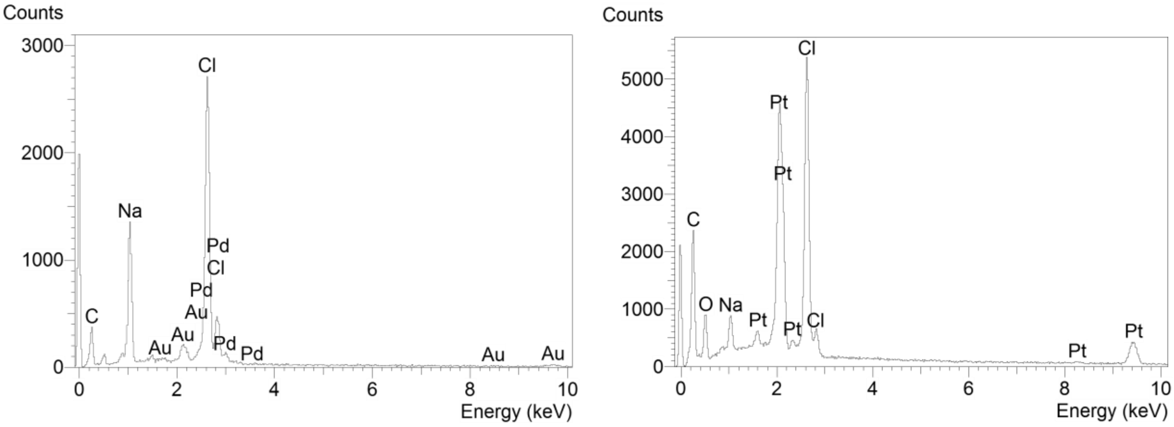

A) B)

Figure S4. X-ray energy-dispersive analysis (EDS) spectra of dark spots of A) Pd-H-Ch and B) Pt-H-Ch.

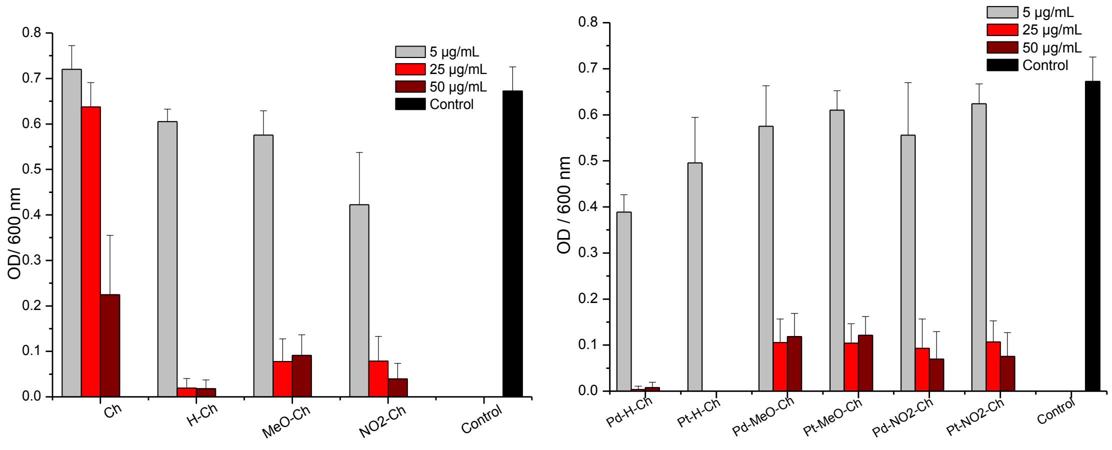

A) B)

Figure S5. Dose response relationship for the inhibitory effect of A) chitosan and Schiff bases, B) Pd(II) and Pt(II) complexes on the growth of *P.syringae* after 24 h. Three replications were used for each treatment and the experiment was carried out three times, from which standard derivation was calculated.

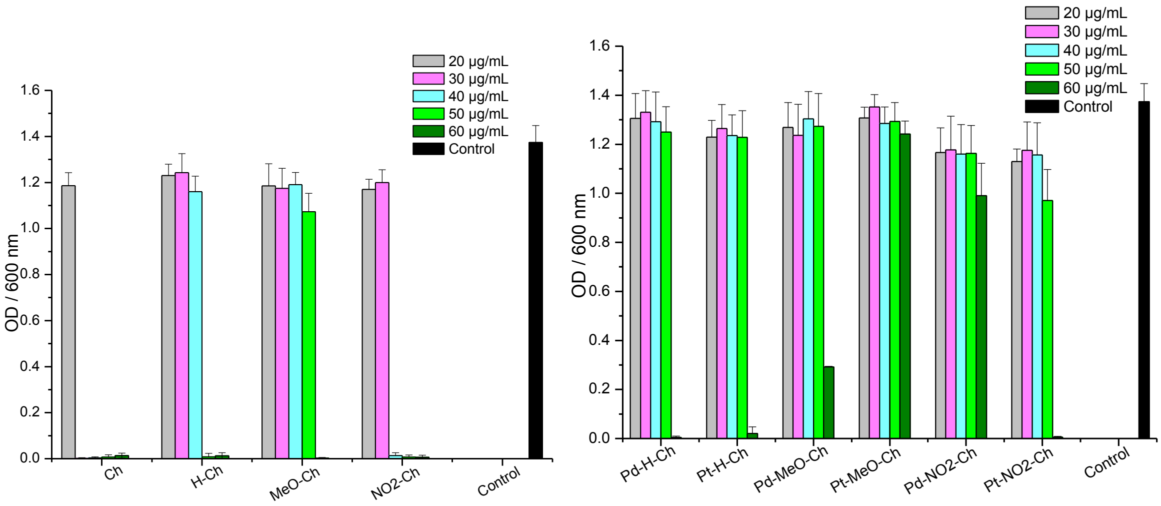

A) B)

Figure S6. Antifungal activity of A) Schiff bases and B) Pd(II), Pt(II) complexes on the growth of *F. graminearum* . Fungal growth was assessed by measuring the optical density of the culture media at 600 nm after 96 h. Three replications were used for each treatment and the experiment was carried out three times, from which standard derivation was calculated.
